# A comparison of barbed continuous suture versus conventional interrupted suture for fascial closure in total hip arthroplasty

**DOI:** 10.1038/s41598-022-07862-5

**Published:** 2022-03-10

**Authors:** Sunhyung Lee, Taehong Kee, Mi Yeon Jung, Pil Whan Yoon

**Affiliations:** Department of Orthopaedic Surgery, Asan Medical Center, University of Ulsan College of Medicine, 43-gil, 88 Olympic-ro, Songpa-gu, Seoul, South Korea

**Keywords:** Outcomes research, Musculoskeletal system, Osteoarthritis

## Abstract

A barbed suture is a self-anchoring knotless suture hypothesized to shorten suture time and reduce the tension point of the wound. The purpose of this study was to compare the barbed suture and the interrupted suture for fascial closure in total hip arthroplasty. We retrospectively reviewed patients who underwent total hip arthroplasty from March 2014 to June 2020. We evaluated 324 cases among 274 patients consisting of 188 males and 86 females. We collected the following data: demographics, time for wound closure, the number of threads used, hemoglobin level, surgical site pain, and wound complications. Variables were analyzed for their association with closure time using multiple regression analyses between the barbed suture (the SFX group) and the interrupted suture (the Vicryl group). Mean closure time was 5.8 min lower and the mean number of sutures used was 2.2 lower in the SFX group versus the Vicryl group (*P* < 0.01 and < 0.01, respectively). There were no statistical intergroup differences in the mean largest hemoglobin drop, the incidence of transfusion, surgical site pain, and the incidence of wound complications. The use of barbed sutures for fascial closure in total hip arthroplasty effectively reduces the surgical time without increasing wound complications.

## Introduction

Interrupted sutures have conventionally been used in fascial closure for total hip arthroplasty (THA). However, tying every knot is a time-consuming procedure, and the tightened knot may impair tissue healing by allowing tissue overlap and interfering with fibroblast proliferation. Bulky knots may be a nidus for infection, and they may extrude through skin weeks after surgery^1^. A barbed suture is a self-anchoring knotless suture and hypothesized to shorten suture time and reduce the tension point of the wound^2^. Since the firstly described by R.A. McKenzie in 1967^3^, barbed suture became commercialized and has been used in the various medical field like general^4–9^, urological^10,11^, plastic^12–14^, and gynecological surgeries^15–19^.

In the field of orthopaedic surgery, several studies on barbed sutures in total knee arthroplasty have been published^20–31^. In most of these, barbed sutures reduced operation time, improved cost-effectiveness, and did not increase the rate of wound complications. However, there is a dearth of published data on the barbed suture technique for THA relative to that for total knee arthroplasty.

In particular, data are lacking on the outcomes of applying barbed sutures for fascial closure in THA. Papers have analyzed the application of barbed sutures to the subcutaneous and subcuticular layers in addition to the fascial layer^20,30,32–36^. However, few have only applied barbed sutures to the fascial layer alone, which makes it difficult to evaluate the suitability of barbed sutures for fascial closure. Inadequate fascial closure causes adverse wound reactions, such as hematoma and oozing, and increases the risk of deep infection, resulting in severe complications.

The purpose of this study was to compare the barbed suture and the interrupted suture for fascial closure in THA. The following factors were assessed in this retrospective analysis of prospectively collected data: (1) closure time; (2) number of threads used; (3) perioperative hemoglobin (Hb) level; (4) Visual Analogue Scale (VAS) for surgical site pain; and (5) complications related with a surgical wound.

## Materials and methods

### Ethical statements

The study was performed following approval from the institutional review board of Asan Medical Center (No. 2020–1531). Informed consent was waived by the institutional review board of Asan Medical Center and all methods were carried out in accordance with relevant guidelines.

### Study population

We performed a retrospective study of prospectively collected data for patients aged 18 years and older who underwent primary THA. The study was performed in accordance with guidelines of institutional review board. The informed consent was waived. All patients were treated by a single surgeon at a single institution, Asan Medical Center, from March 2014 to June 2020. During the period, 997 cases of primary THA were performed at this institution. Among these, 565 cases which were diagnosed with osteonecrosis of the femoral head (ONFH) were included in this study. To minimize the factors affecting the surgical procedure time and wound complications, cases with the following conditions were excluded: (1) post-traumatic ONFH (31 cases); (2) THAs performed by an approach other than the posterolateral approach (8 cases); (3) prior surgery for ONFH (39 cases); (4) possible bleeding tendency, e.g., Child–Pugh class B, C, or taking anticoagulants (28 cases); (5) cable fixation during the surgery (16 cases); (6) no intra-operative radiograph (53 cases); and (7) skin closure with a staple (44 cases). Cases with follow-up loss before postoperative 3 months (19 cases), osteogenesis imperfecta (1 case), use of closed suction drainage (1 case), and stem exchange after real implant insertion (1 case) were also excluded. Finally, 324 cases of THA surgeries among 274 patients were evaluated, consisting of 188 males and 86 females with a mean age of 51.6 years (range, 18–84 years). Baseline demographic and medical history data were collected from the electronic medical record, and the Charlson Comorbidity Index (CCI)^37^ was calculated for each patient.

### Surgical technique

All analyzed cases were diagnosed with ONFH and underwent THA via the posterolateral approach. Femoral procedures were done after the acetabular procedure including the insertion of a real acetabular cup and a liner. After serial rasping on the femoral side, an intraoperative radiograph of the pelvis was taken to evaluate the cup position and the size of the femoral component. Insertion of real femoral component, manual test for joint stability, irrigation, capsulotomy site repair, piriformis and short external rotator muscles repair, fascial repair, and suture for subcutaneous and skin layer was done serially. Closed suction drainage was not used in routine THA procedures.

Except for fascial closure, the closure process was performed by the same suture materials and techniques in all cases. Number #2 triclosan-coated glycolide-lactide absorbable suture (Vicryl Plus Antibacterial Suture, Ethicon; Johnson & Johnson, USA) was used to repair the capsulotomy site, piriformis muscle, and short external rotator muscles, and to close the subcutaneous layer. Number 2-0 violet braided polyglactin 910 absorbable suture (Vicryl Plus, Ethicon) was used for closing the subcuticular layer. For skin closure, adhesive mesh with glue (Prineo, Ethicon) was used.

**Figure 1:**
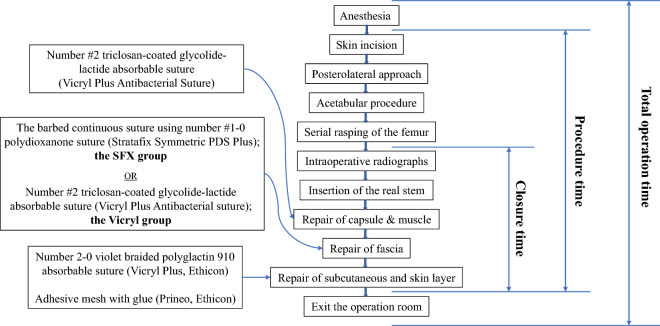
A flowchart for the surgical procedure, suture methods, and time intervals.

For fascial closure, either the barbed continuous suture using number #1–0 polydioxanone suture (Stratafix Symmetric PDS Plus, Ethicon) or the interrupted suture using number #2 triclosan-coated glycolide-lactide absorbable suture (Vicryl Plus Antibacterial suture, Ethicon) was used. The interrupted suture was used before October 2018, whereas the barbed suture was used after October 2018 (Fig. 1).

### Outcome measures

From the surgical record, the number of threads used and the closure type for fascia were addressed. Time for the start of anesthesia, skin incision, intraoperative radiograph, skin closure and exit from the operation room were also collected. Using these data, three different time intervals were calculated. “Closure time” was defined as the time from the intraoperative radiograph to the initiation of skin closure. In accordance with this definition, the closure time included insertion of real femoral component, irrigation, repair for capsule, muscle, and fascia, and closure for subcutaneous tissue. “Procedure duration” was defined as the time from the skin incision to the initiation of skin closure. “Total operation time” was defined as the time from the start of anesthesia to exit from the operation room (Fig. 1).

Patients were routinely discharged on the fifth day of the surgery, and routine visits were scheduled at postoperative six weeks and three months. Hb levels were checked before the surgery, on the first, third, fifth days, and six weeks after the surgery. The level of the largest Hb drop during the admission days was measured. Whether blood transfusion was performed during hospital days were also recorded. VAS for pain in the affected hip was assessed preoperatively and postoperatively, and improvement in VAS was calculated. Wound complications based on the criteria of Healy et al.^38^ were analyzed during the hospital stay and at the outpatient clinic. Readmission and reoperation were defined as any event related to THA that required readmission or reoperation.

### Statistical analysis

Two-tailed Student t-tests or Mann–Whitney tests were used to evaluate continuous outcomes, with a 0.05 alpha-level for statistical significance, the latter which was used when normality could not be assumed. To assess dichotomous variables, the chi-square test was used unless cells with a frequency of five or less exceeded 20%, in which case Fisher’s exact test was performed. To adjust for six confounders (wound closure type, age, gender, smoking history, BMI, and CCI) that may be associated with closure time or wound complications, linear and logistic multiple regression analysis was conducted for continuous and dichotomous dependent variables, respectively. All analyses were conducted using R software (version 4.0.3).

## Results

Of 324 cases, fascial closure was done with a barbed continuous suture in 126 cases (SFX group) and with an interrupted suture in 198 cases (Vicryl group). The mean age was 3.3 years younger in the SFX group (*P* = 0.04), and the proportion of current smokers was higher in the SFX group (30.2% versus 16.2% in the Vicryl group, *P* = 0.01). Other demographic factors such as gender, body mass index (BMI), diabetes mellitus**,** CCI, and preoperative use of iron medication showed no significant difference between the two groups (Table 1).

**Table 1:** Patient demographics.

Variables	SFX group (n = 126)	Vicryl group (n = 198)	Total (n = 324)	*p *value
**Age (years, mean ± SD)**	48.9 ± 14.1	52.2 ± 13.4	50.9 ± 13.8	**0.04**^a^
**Gender (n, %)**				
Male	89 (70.6%)	131 (66.2%)	220 (68.0%)	
Female	37 (29.4%)	67 (33.8%)	104 (32.0%)	0.47^b^
Total	126 (100%)	198 (100%)	324 (100%)	
BMI (kg/m^2^, mean ± SD)	23.9 ± 3.6	23.9 ± 3.5	23.9 ± 3.5	0.93^a^
**Smoking (n, %)**				
Never	59 (46.8%)	103 (52.0%)	162 (50.0%)	
Ex-smoker	29 (23.0%)	63 (31.8%)	92 (28.4%)	**0.01**^b^
Current smoker	38 (30.2%)	32 (16.2%)	70 (21.6%)	
Total	126 (100%)	198 (100%)	324 (100%)	
**Diabetes mellitus (n, %)**	15 (11.9%)	11 (5.6%)	26 (8.0%)	0.07^b^
**CCI score (mean ± SD)**	0.7 ± 1.3	0.5 ± 1.2	0.6 ± 1.2	0.43^a^
**Preoperative iron medication (n, %)**				
No	125 (99.2%)	196 (99.0%)	321 (99.1%)	
Yes	1 (0.8%)	2 (1.0%)	3 (0.9%)	1.00^b^
Total	126 (100%)	198 (100%)	324 (100%)	
The second THA for staged THAs (n, %)	11 (8.7%)	29 (14.6%)	40 (12.3%)	0.12^b^

Significant values are in bold.

SD, Standard deviation; BMI, body mass index; CCI, Charlson Comorbidity Index.

^a^By independent t-test.

^b^By chi-square test.

Forty-nine patients underwent staged total hip arthroplasties, whereas no simultaneous bilateral total hip arthroplasties were conducted in this study. There was no statistical difference between the two groups in the rate of patients who underwent staged total hip arthroplasties (8.7% in the SFX group and 14.6% in the Vicryl group, *P* = 0.11) (Table 1). No overall wound complications were observed in these patients.

The time for all three evaluated operating intervals for THA was statistically lower in the SFX group: mean closure time was lower by 5.8 min (*P* < 0.01), mean procedure duration by 8.1 min (*P* < 0.01), and mean total operation time by 8.1 min (*P* < 0.01). The mean number of #2 Vicryl sutures used for closure was 2.2 lower in the SFX group compared with the Vicryl group (*P* < 0.01) (Table 2).

**Table 2:** Surgical time and the number of threads used according to suture type of THA patients.

Variables	SFX group (n = 126)	Vicryl group (n = 198)	*p *value
Closure time (min, mean ± SD)	46.5 ± 10.5	52.3 ± 10.4	< **0.01**^a^
Procedure duration (min, mean ± SD)	74.8 ± 12.8	82.9 ± 14.5	< **0.01**^a^
Total operation time (min, mean ± SD)	130.7 ± 17.0	138.8 ± 18.3	< **0.01**^a^
Number of used Vicryl (n, mean ± SD)	3.1 ± 0.5	5.3 ± 1.4	< **0.01**^a^

Significant values are in bold.

^a^By independent t-test.

SD, Standard deviation; Closure time, from intraoperative X-ray to skin closure; Procedure duration, from incision to skin closure; Total operation time, from anesthesia to exiting operation room.

Intergroup differences in the mean of the largest Hb drop during the hospitalization were not statistically significant (− 2.5 g/dl in the SFX group versus − 2.6 g/dl in the Vicryl group, *P* = 0.37). In the SFX group, the mean Hb level was 12.8 g/dl preoperatively and 13.5 g/dl at postoperative 6 weeks. These showed no statistical significance with those of Vicryl group, respectively; 13.1 g/dl preoperatively (*P* = 0.12), and 13.4 g/dl at postoperative 6 weeks (*P* = 0.60). The number of cases that had undergone blood transfusion during the admission days was 17 (13.5%) in the SFX group and 44 (22.2%) in the Vicryl group. There was no statistical difference between the two (*P* = 0.07). Improvement in VAS for hip pain also showed no statistically significant difference between the two groups (*P* = 0.07) (Table 3).

**Table 3:** Clinical outcomes according to suture type of THA patients.

Variable	SFX group (n = 126)	Vicryl group (n = 198)	*p *value
**Largest Hb drop during the hospital days (g/dl, mean ± SD)**	− 2.5 ± 1.3	− 2.6 ± 1.5	0.37^a^
**Hb level in preoperative status (g/dl, mean ± SD)**	12.8 ± 2.0	13.1 ± 1.8	0.12^a^
**Hb level in postoperative 6 weeks (g/dl, mean ± SD)**	13.5 ± 1.6	13.4 ± 1.4	0.60^a^
**Cases with blood transfusion (n, %)**			
No	109 (86.51%)	154 (77.78%)	
Yes	17 (13.49%)	44 (22.22%)	0.07^b^
Total	126 (100%)	198 (100%)	
Improvement in VAS (mean ± SD)	− 0.4 ± 0.9	− 0.3 ± 0.7	0.07^a^

Hb, hemoglobin; SD, standard deviation; VAS, Visual Analogue Scale.

^a^By independaent t-test.

^b^By chi-square test.

There were 4 cases with wound complications, 1 in the SFX group and 3 in the Vicryl group, with no statistical differences in complication rates between groups (*P* = 1.00). Brief patient information for each case was as follows: (1) A 31-year-old female patient in the SFX group showed oozing on the surgical site combined with stitch abscess at postoperative 6 weeks. Two times of debridement and wound revision was done. There was a substantial amount of liquified hematoma in the subcutaneous layer. Dehiscence of the fascial layer, though, was not observed during the two operations. Coagulase-negative staphylococcus was identified from the stitch abscess, but no bacteria were identified in the subcutaneous layer; (2) A 67-year-old female patient in the Vicryl group complained of wound dehiscence of the skin layer at 17 days from the index surgery. Wound revision was done at postoperative 18 days; (3) A 66-year-old male patient in the Vicryl group complained of discomfort at the surgical site. The area which was presumed to be a hematoma site was firmly palpated. Discomfort disappeared by conservative treatment; and (4) A 67-year-old female patient in the Vicryl group showed stitch abscess at postoperative 6 weeks. A minimal amount of fluid was naturally drained and the wound was healed without other complications. There were no findings suggesting deep surgical site infection or periprosthetic joint infection in the aforementioned cases (Table 4).

**Table 4:** Wound related complications.

	Closure type	Type of complication	Number of reoperation
Case 1	SFX	Oozing, stitch abscess, and hematoma	2
Case 2	Vicryl	Wound dehiscence	1
Case 3	Vicryl	Hematoma	0
Case 4	Vicryl	Stitch abscess	0

SFX, fascial closure with Stratafix; Vicryl, fascial closure with Vicryl.

Multiple regression analysis showed wound closure type and BMI each had a statistically significant effect on closure time of the THA patients (*P* < 0.01 and *P* = 0.04, respectively). Cases with the barbed suture or low BMI required shorter closure time, although BMI was not statistically different between the two groups in this study. Closure type did not have a statistically significant effect on the occurrence of wound complications related to THA (*P* = 0.47) (Table 5).

**Table 5:** Multiple regression analysis for the closure time and wound complications.

Variables	Univariable		Multivariable	
	Beta or crude OR (95% CI)	*p *value	Beta or adjusted OR (95% CI)	*p *value
**Closure time**^**a**^				
Wound closure type	− 5.78 (− 8.02 to − 3.44)	** < 0.01**	− 5.81 (− 8.17 to − 3.44)	** < 0.01**
Age	0.01 (− 0.08 to 0.09)	0.84	− 0.03 (− 0.11 to 0.06)	0.52
Gender	− 0.57(− 3.09 to 1.95)	0.66	− 1.19 (− 4.00 to 1.63)	0.41
History of smoking	− 0.79 (− 2.26 to 0.69)	0.29	− 0.81 (− 2.46 to 0.84)	0.34
BMI	0.34 (0.01 to 0.67)	**0.04**	0.35 (0.02 to 0.68)	**0.04**
CCI	0.14 (− 0.82 to 1.11)	0.77	0.34 (− 0.60 to 1.28)	0.47
**Wound complications**^**b**^				
Wound closure type	0.52 (0.03 to 4.11)	0.57	0.36 (0.01 to 5.33)	0.51
Age	1.04 (0.97 to 1.14)	0.32	1.06 (0.97 to 1.21)	0.27
Gender	6.51 (0.82 to 132.39)	0.11	24.51 (1.04 to 1825.27)	0.09
History of smoking	0.68 (0.12 to 2.39)	0.59	4.08 (0.38 to 58.61)	0.25
BMI	1.39 (1.06 to 1.86)	**0.02**	1.57 (1.12 to 2.55)	**0.03**
CCI	1.28 (0.64 to 1.89)	0.30	1.39 (0.72 to 2.30)	0.20

Significant values are in bold.

## Discussion

This study suggests that utilization of barbed suture materials can reduce surgical time and the number of threads used for THA. These results were observed without an increase in surgical site wound complications. These results are consistent with published studies on total knee arthroplasties^20–31^. The barbed suture technique applied only to the fascial layer in THA has little been studied except for one study^39^. Most prior studies addressed barbed sutures for both the fascia and subcutaneous layer^20,30,32–36^, subcutaneous layer only^40^, or subcuticular layer only^41–43^. Inconsistent application of the barbed sutures during wound closure creates difficulty in comparing the results of these studies. The meta-analysis on these studies^20,44,45^ also has limitations for the heterogeneity of the target studies.

Sundaram et al.^39^ published a randomized controlled trial for the method of suture technique in the fascial layer of 60 THA patients. The study showed, as in our study, a statistically significant reduction of closure time by 5 min for the barbed suture group, and no intergroup difference in wound complications. However, the study was limited by a relatively small sample size (30 patients per group), which is not ideal for evaluating rare operative complications. Our study indicated that the use of barbed sutures can improve the efficiency of THA by reducing the surgical time by 5–8 min with fewer threads than required for conventional methods. The most challenging issue in the medical market is always how to efficiently distribute limited medical resources. Given that time is a limited medical resource to be allocated, efficiency in performing THA is the value that we must increase. In this respect, the use of barbed sutures, which can reduce overall operation time, is a good option for THA surgery.

In addition to time savings, the number of threads used was 2.2 less in the SFX group than in the Vicryl group. Considering that the length of the Vicryl (Ethicon) suture is 70 cm and that of the Stratafix (Ethicon) is 45 cm, barbed sutures allow suture of the surgical wound with a shorter length of threads. This furthers the efficient allocation of medical resources.

Utilization of barbed sutures in THA does not appear to worsen the surgical outcome of THA. By a cadaveric experiment by Nett et al.^46^, a barbed continuous suture is more watertight than a conventional interrupted suture. Although watertightness was not evaluated in this study, there was no difference in Hb level drop, transfusion rate, or occurrence of operative site hematoma between the SFX and Vicryl groups. Of the observed wound complications in this study, only hematomas are associated with inadequate fascial closure. Hematoma occurred in one case each in both patient groups. Although a barbed suture is expected to reduce surgical infection^1,2^, deep infection, which can be caused by inadequate closure of the fascial layer, did not occur in either group in this study. Since the surgical site for THA was a clean surgical wound, deep infections were unlikely to occur unless an external source of infection was inoculated. This may be why deep infection was not observed in this study. Therefore, in our study the barbed suture enabled more efficient wound closure without increasing wound complications.

There are some limitations to this study, including those inherent to study design. First, all patients included in this study were Asians, given the location of the institution was South Korea. However, THA procedures is not expected to vary significantly by patients’ race. Second, the SFX group was on average 3.3 years younger and the proportion of smokers was about 2 times higher than the Vicryl group. The authors believe that the age difference of about 3 years does not bear clinical significance. Further, the statistically significant effect of closure type persisted after multiple regression analysis, which included age and smoking as possible confounders. Considering that the smoking adversely affects surgical wound healing, it should be noted when interpreting the result of this study that the smoking rate was about twice as high in the SFX group. Third, the exact time for fascial closure was not assessed, as it was not routinely recorded in this institution. However, the surgical procedure in this study was standardized as much as possible by including only ONFH patients who had undergone THA by the posterolateral approach and excluding cases with factors likely to affect operation time such as bleeding tendency, cable fixation, or prior surgery of the affected hip. The three different time intervals which was calculated in this study showed similar decreases in the SFX group. Through this, the fascial closure time can be indirectly estimated. Finally, although the use of barbed sutures in THA increases surgical efficiency, further research is needed to ascertain its cost effectiveness. The price of the thread used in the SFX group (Stratafix, Ethicon) was about 25 $ and that in the Vicryl group (Vicryl, Ethicon) was about 5 $ in South Korea. This study showed that the use of barbed sutures reduces the number of threads used by 2.2 on average; nonetheless, if price is the only consideration, the SFX group is at a disadvantage. Though, the value of sutures should be quantified in the context of all potential cost offsets, like time savings given that the time is the limited medical resources. Such calculations are challenging in the light of the complexity and variability of the medical system by institutions and countries.

## Conclusions

This study suggests that the use of barbed sutures for fascial closure in THA effectively reduces the surgical time as well as the number of threads without increasing the wound complications.

## Data Availability

The data analyzed during the current study are not publicly available due to the policy of the institutional review board of Asan Medical Center but are available from the corresponding author on reasonable request.

## References

[1] Murtha AP (2006). Evaluation of a novel technique for wound closure using a barbed suture. Plast. Reconstr. Surg..

[2] Dennis C (2016). Suture materials: Current and emerging trends. J. Biomed. Mater. Res. A.

[3] McKenzie AR (1967). An experimental multiple barbed suture for the long flexor tendons of the palm and fingers. Preliminary report. J. Bone Jt. Surg. Br..

[4] Demyttenaere SV (2009). Barbed suture for gastrointestinal closure: a randomized control trial. Surg. Innov..

[5] Manigrasso M (2019). Barbed suture and gastrointestinal surgery. a retrospective analysis. Open Med. (Wars).

[6] Pennestri F (2019). Barbed vs conventional sutures in bariatric surgery: a propensity score analysis from a high-volume center. Updates Surg..

[7] Ferrer-Marquez M (2020). Use of barbed suture for the closure of enterocolotomy after laparoscopic right hemicolectomy with intracorporeal anastomosis. A prospective descriptive study. Cir. Esp..

[8] Fujiwara S, Kaino K, Iseya K, Koyamada N (2020). Laparoscopic subtotal cholecystectomy for difficult cases of acute cholecystitis: a simple technique using barbed sutures. Surg. Case Rep..

[9] Wiggins T (2020). Benefits of barbed suture utilisation in gastrointestinal anastomosis: a systematic review and meta-analysis. Ann. R. Coll. Surg. Engl..

[10] Lin Y (2019). The application of barbed suture during the partial nephrectomy may modify perioperative results: a systematic review and meta-analysis. BMC Urol..

[11] Zhan H (2019). The self-retaining barbed suture for parenchymal repair in laparoscopic partial nephrectomy: a systematic review and meta-analysis. Surg. Innov..

[12] Cortez R (2015). Barbed sutures and wound complications in plastic surgery: An analysis of outcomes. Aesthet. Surg. J..

[13] Paul MD (2013). Barbed sutures in aesthetic plastic surgery: Evolution of thought and process. Aesthet. Surg. J..

[14] Shermak MA (2013). The application of barbed sutures in body contouring surgery. Aesthet. Surg. J..

[15] Zayed MA (2019). Barbed sutures versus conventional sutures for uterine closure at Cesarean section; a randomized controlled trial. J. Matern. Fetal Neonatal. Med..

[16] Greenberg JA, Einarsson JI (2008). The use of bidirectional barbed suture in laparoscopic myomectomy and total laparoscopic hysterectomy. J. Minim. Invasive Gynecol..

[17] Lopez CC (2019). Barbed suture versus conventional suture for vaginal cuff closure in total laparoscopic hysterectomy: randomized controlled clinical trial. J. Minim. Invasive Gynecol..

[18] Pepin K (2020). Reproductive outcomes following use of barbed suture during laparoscopic myomectomy. J. Minim. Invasive Gynecol..

[19] Grin L (2019). Barbed versus conventional suture for uterine repair during caesarean section: a randomized controlled study. J. Obstet. Gynaecol. Can..

[20] Borzio RW, Pivec R, Kapadia BH, Jauregui JJ, Maheshwari AV (2016). Barbed sutures in total hip and knee arthroplasty: what is the evidence? A meta-analysis. Int. Orthop..

[21] Chan VWK, Chan PK, Chiu KY, Yan CH, Ng FY (2017). Does barbed suture lower cost and improve outcome in total knee arthroplasty? a randomized controlled trial. J. Arthroplasty.

[22] Eickmann T, Quane E (2010). Total knee arthroplasty closure with barbed sutures. J. Knee Surg..

[23] Faour M (2018). The role of barbed sutures in wound closure following knee and hip arthroplasty: a review. J. Knee Surg..

[24] Gililland JM (2014). Barbed versus standard sutures for closure in total knee arthroplasty: a multicenter prospective randomized trial. J. Arthroplasty.

[25] Gililland JM, Anderson LA, Sun G, Erickson JA, Peters CL (2012). Perioperative closure-related complication rates and cost analysis of barbed suture for closure in TKA. Clin. Orthop. Relat. Res..

[26] Levine BR, Ting N, DellaValle CJ (2011). Use of a barbed suture in the closure of hip and knee arthroplasty wounds. Orthopedics.

[27] Maheshwari AV (2015). Barbed sutures in total knee arthroplasty: are these safe, efficacious, and cost-effective?. J. Knee Surg..

[28] Malhotra R, Jain V, Kumar V, Gautam D (2017). Evaluation of running knotless barbed suture for capsular closure in primary total knee arthroplasty for osteoarthritis-a prospective randomized study. Int. Orthop..

[29] Sah AP (2015). Is there an advantage to knotless barbed suture in TKA wound closure? A randomized trial in simultaneous bilateral TKAs. Clin. Orthop. Relat. Res..

[30] Smith EL, DiSegna ST, Shukla PY, Matzkin EG (2014). Barbed versus traditional sutures: closure time, cost, and wound related outcomes in total joint arthroplasty. J. Arthroplasty..

[31] Sun, C., et al. Barbed sutures in total knee arthroplasty: a meta-analysis of randomized-controlled trials. *J. Knee Surg*. (2020). 10.1055/s-0040-1710373 32462647

[32] Li R (2018). A modified strategy using barbed sutures for wound closure in total joint arthroplasty: a prospective, randomized, double-blind, self-controlled clinical trial. Med. Sci. Monit..

[33] Sah AP (2021). A prospective, randomized evaluation of the quality of wound closure with barbed versus standard suture after total joint arthroplasty. Orthopedics.

[34] Serrano Chinchilla P, Gamba C, Leon Garcia A, Tey Pons M, Marques Lopez F (2021). Use of barbed suture in total hip prosthesis. Prospective randomized study. Rev. Esp. Cir Ortop. Traumatol. (Engl. Ed.).

[35] Sutton N, Schmitz ND, Johnston SS (2018). Comparing outcomes between barbed and conventional sutures in patients undergoing knee or hip arthroplasty. J. Comp. Eff. Res..

[36] Ting NT, Moric MM, Della Valle CJ, Levine BR (2012). Use of knotless suture for closure of total hip and knee arthroplasties: a prospective, randomized clinical trial. J. Arthroplasty.

[37] Charlson ME, Pompei P, Ales KL, MacKenzie CR (1987). A new method of classifying prognostic comorbidity in longitudinal studies: development and validation. J. Chronic Dis..

[38] Healy WL (2016). Complications of total hip arthroplasty: standardized list, definitions, and stratification developed by the hip society. Clin. Orthop. Relat. Res..

[39] Sundaram, K., et al. Barbed sutures reduce arthrotomy closure duration and suture utilisation compared to interrupted conventional sutures for primary total hip arthroplasty: a randomised controlled trial. *Hip Int*. 1120700020911891 (2020). 10.1177/1120700020911891 32188284

[40] Thacher RR, Herndon CL, Jennings EL, Sarpong NO, Geller JA (2019). The impact of running, monofilament barbed suture for subcutaneous tissue closure on infection rates in total hip arthroplasty: a retrospective cohort analysis. J. Arthroplasty.

[41] Knapper TD (2019). Barbed sutures versus staples for closure in total hip arthroplasty using wound ooze as a primary outcome measure: A prospective study. J. Orthop. Surg..

[42] Patel RM, Cayo M, Patel A, Albarillo M, Puri L (2012). Wound complications in joint arthroplasty: comparing traditional and modern methods of skin closure. Orthopedics.

[43] Roumeliotis L, Graham NM (2019). Barbed suture and glue in skin closure during lower limb arthroplasty: reduced delayed discharge due to wound exudate. J. Wound Care.

[44] Han Y (2018). The efficacy and safety of knotless barbed sutures in total joint arthroplasty: a meta-analysis of randomized-controlled trials. Arch. Orthop. Trauma Surg..

[45] Snyder MA, Chen BP, Hogan A, Wright GWJ (2021). Multilayer watertight closure to address adverse events from primary total knee and hip arthroplasty: a systematic review of wound closure methods by tissue layer. Arthroplasty Today.

[46] Nett M, Avelar R, Sheehan M, Cushner F (2011). Water-tight knee arthrotomy closure: comparison of a novel single bidirectional barbed self-retaining running suture versus conventional interrupted sutures. J. Knee Surg..

